# Comparison of Serological and Molecular Assays for *Bartonella* Species in Dogs with Hemangiosarcoma

**DOI:** 10.3390/pathogens10070794

**Published:** 2021-06-23

**Authors:** Erin Lashnits, Pradeep Neupane, Julie M. Bradley, Toni Richardson, Ricardo G. Maggi, Edward B. Breitschwerdt

**Affiliations:** 1 Department of Medical Sciences, School of Veterinary Medicine, University of Wisconsin, Madison, WI 53713, USA; lashnits@wisc.edu; 2 Intracellular Pathogens Research Laboratory, Comparative Medicine Institute, College of Veterinary Medicine, North Carolina State University, Raleigh, NC 27606, USA; pneupan@ncsu.edu (P.N.); jmbradl3@ncsu.edu (J.M.B.); tgrichar@ncsu.edu (T.R.); rgmaggi@ncsu.edu (R.G.M.); 3 Department of Clinical Sciences, College of Veterinary Medicine, North Carolina State University, Raleigh, NC 27606, USA

**Keywords:** diagnostic testing, ddPCR, PCR, serology, sensitivity, specificity, tissue biopsy, latent class analysis

## Abstract

Currently, a gold standard diagnostic test for *Bartonella* infection in dogs is lacking. This represents a critical limitation for the development and evaluation of new diagnostic tests, as well as for the diagnosis of, and research on, bartonellosis in dogs. This retrospective observational study aims to compare the results of commonly performed and newly-reported *Bartonella* spp. diagnostic tests in banked clinical specimens from 90 dogs with hemangiosarcoma (HSA) using composite reference standard (CRS) and random effects latent class analysis (RE-LCA) techniques. Samples from each dog were tested using six serological or molecular diagnostic assays, including indirect fluorescent antibody (IFA) and Western blot (WB) for the detection of antibodies in serum, and qPCR and droplet digital PCR (ddPCR) in blood and fresh frozen tissue biopsy samples (mainly splenic HSA tumors and histopathologically normal spleen or skin/adipose tissue). *Bartonella* infection prevalence was estimated to be 78% based on the CRS (parallel testing with all six assays), and 64% based on the RE-LCA model. The assay with the highest diagnostic accuracy was qPCR performed on fresh frozen tissue biopsy samples (sensitivity: 94% by RE-LCA and 80% by CRS; specificity: 100%). When comparing newly-reported to traditional *Bartonella* diagnostic assays, ddPCR was more sensitive for the detection of *Bartonella* DNA than qPCR when testing blood samples (36% vs. 0%, *p* < 0.0001). Dogs that were positive on serological assays alone with negative molecular assays were highly unlikely (<3%) to be classified as infected by the RE-LCA model. These data indicate that *Bartonella* spp. DNA can be PCR amplified from fresh frozen tissues from a majority of dogs with HSA using both qPCR and ddPCR, supporting the use of these methods for future controlled studies comparing the prevalence of *Bartonella* spp. DNA in the tissue of dogs with HSA to that of unaffected controls.

## 1. Introduction

Documentation of zoonotic bartonelloses in humans in the 1990s predated the identification of the first case of bartonellosis in a dog by only a few years [1,2,3]. The ongoing discovery of new *Bartonella* spp., seventeen of which have been associated with disease in dogs or humans, continues to present challenges to diagnosticians, clinicians, and patients in both human and veterinary medicine [4,5,6,7]. In human cases of zoonotic bartonellosis, diagnosis has historically relied on serology, culture from blood or tissue, and visualization of bacteria with silver stains in lymph node biopsies from patients with suspected Cat Scratch Disease (CSD) [8]. Initially developed to assess *B. henselae* seroreactivity in patients with CSD, an indirect fluorescent antibody (IFA) assay subsequently became the reference standard for the diagnosis of bartonellosis (the best method available), and the test to which newly developed assays were compared [9,10]. Advances in serological methods and the advent of molecular-based assays have facilitated the diagnosis of bartonellosis in some clinically suspected cases; however, there remains uncertainty regarding the diagnostic accuracy of currently available tests for *Bartonella* spp. infection, particularly in patients with nonspecific, chronic symptoms, and in patients with what have been historically considered atypical manifestations of CSD [4,11,12].

Because of the fastidious growth conditions required by *Bartonella* spp., the sensitivity of culture is estimated to be only 20–30%, even with specialized culture procedures and media (5% CO_2_, 35–37 °C, various specialized media), but varies substantially depending on the *Bartonella* spp., clinical presentation, tissue used for culture, and antibiotic history of the patient, among other variables [4,13,14,15]. PCR amplification of *Bartonella* spp. DNA from blood or tissue specimens, thought to improve diagnostic sensitivity, is now routinely performed by many commercial diagnostic laboratories. Clinically, PCR sensitivity depends not only on laboratory factors like the choice of PCR primers and *Bartonella* gene targets, the equipment and methodological considerations, but also on the clinical presentation (illness duration and other factors) and sample type (blood, other body fluid, tissue biopsy, etc.) [15,16,17]. With so many factors influencing the accuracy of PCR, a broad range of PCR sensitivity has been reported (33–92%), even when testing solely patients with prototypical diseases caused by *Bartonella* spp. infection, such as endocarditis and CSD [14,18]. A true gold standard test is defined by a sensitivity and specificity of 100%, so the current human reference standard of serology is far from that. Studies investigating the sensitivity and specificity of serology in *Bartonella* spp. infection have mainly focused on patients with acute CSD or culture-negative endocarditis (presumably a more chronic infection with the eventual localization of bacteria to the heart valve). Similar to reports on PCR, these studies of *Bartonella* spp. serology report highly variable sensitivity (20–90%), though specificity has been better (93–98%) [4,11,19,20,21,22,23]. The accuracy of serological testing in chronic bartonellosis or in manifestations other than acute CSD or endocarditis has not been rigorously assessed, though serology is thought to have poor sensitivity in these “atypical” cases based on observations that people with documented *Bartonella* spp. DNA in their bloodstream or tissues are often seronegative [24,25,26,27,28]. Conceptually, serology also may have poor sensitivity due to the potential for antigenic switching and immune evasion, and poor specificity for active infection since seroreactivity may persist after exposure, despite effective immunological or antibiotic elimination of the infection [18,20,29]. Indeed, finding false negative serology in actively infected dogs can be common with other infectious and vector borne diseases, particularly those with an intracellular lifestyle such as brucellosis, leishmaniasis, or fungal diseases [30,31,32,33,34,35]. Lack of specificity of IFA for active infection caused by *Bartonella* spp. is also evidenced by reports of *Bartonella* seroreactivity in apparently healthy asymptomatic people worldwide [36,37,38].

Challenges to definitively diagnose bartonelloses in humans are, unsurprisingly, recapitulated in companion animal veterinary medicine. Bartonellosis in dogs is found worldwide and associated with multiple acute or chronic clinical manifestations including endocarditis, pyogranulomatous inflammatory disease, vasoproliferative lesions, and nonspecific signs such as fever, lymphadenopathy, or uveitis [7]. As in humans, there are numerous species of *Bartonella* reported in dog infections, including most commonly *B. henselae*, *B. vinsonii* subsp. *berkhoffii*, and *B. koehlerae* [39,40]. For zoonotic *Bartonella* spp., accurate diagnosis of *Bartonella* infection in dogs as sentinel species is of paramount importance to informing our evolving understanding of *Bartonella* spp. transmission and the spectrum of disease manifestations attributable to this genus of bacteria [41,42]. There is currently no accepted gold standard test–or test free from error–for the diagnosis of bartonellosis in dogs, either for clinical cases or for use in epidemiologic or clinical research [7,43]. A limited number of studies have investigated the diagnostic accuracy of commonly used *Bartonella* assays in naturally infected dogs [44,45,46]. PCR sensitivity when testing blood, compared to tissue as the specimen source, or when compared to enrichment blood or tissue culture using the *Bartonella* α-proteobacteria growth medium (BAPGM) platform, appears particularly poor, with estimates ranging from 0% up to approximately 50% [46,47,48,49]. Serology has been similarly problematic, with sensitivity estimates ranging from less than 40% up to approximately 60% in naturally infected dogs, despite sensitivities of 100% in experimentally infected dogs [44,46,50,51]. Additionally, multiple studies have suggested a poor correlation between the species/strain IFA seroconversion and *Bartonella* species/strain based on PCR in naturally infected dogs, despite accurate species/strain seroconversion in experimentally infected dogs [44,45,46,47,49,50]. In the absence of a gold standard diagnostic test, estimates of specificity are difficult to obtain in naturally infected dogs. However IFA specificity has been estimated at over 85% when compared to a panel of previously performed diagnostic tests [44]. Additionally, among a large sample of over 15,000 North American dogs suspected of vector-borne disease, only 4% were *Bartonella* spp. seroreactive, suggesting that seroreactivity against *Bartonella* spp. is fairly uncommon even in dogs with potential for vector exposure [39]. Despite the commercial availability of *Bartonella* serology and PCR through many veterinary diagnostic laboratories, diagnostic accuracy metrics are not well established, mainly due to limitations shared with human diagnostic testing modalities–specifically the use of imperfect reference standards for “gold standard” comparisons [44,52].

Because of these historical and current diagnostic limitations, recent efforts have been directed at ongoing improvements in serological and molecular assays that are applicable to both companion animals and human patients [53,54,55]. Though available commercially for over 20 years, validation of interpretation criteria for WB serodiagnosis of bartonelloses in dogs was only recently published [56,57]. Using a combination of naturally and experimentally infected dogs, sensitivity and specificity of WB was estimated at 53% and 96% respectively [56]. In addition, to improve upon current molecular diagnostics, droplet digital PCR (ddPCR)—a technology originally developed to precisely estimate DNA copy number–has been adapted to the detection of rare or low abundance pathogen DNA in a background of abundant host DNA, and applied to the diagnosis of bartonellosis. The analytical and diagnostic validation of ddPCR for detection of *Bartonella* spp. DNA in human blood samples was recently published, with an estimated sensitivity between 50–75% depending on the reference standard, and estimated specificity of 99% [28,58]. This method has not previously been reported for use in companion animal species.

There has been extensive research into statistical methods to evaluate diagnostic accuracy of imperfect tests when no gold standard reference test exists [59,60,61]. Historically, a composite reference standard (CRS) could be defined using a combination of imperfect tests as a reference, but this method relies heavily on the utility of each test and the correct classification of a subject [62,63]. Another approach, known as latent class analysis (LCA), uses probabilistic modeling to classify patients into disease states (the so-called latent variable, here infected or uninfected) based on the observed results of their imperfect diagnostic tests. In LCA, the model-based classification is then used to estimate diagnostic accuracy parameters (sensitivity and specificity), even in the absence of a gold standard.

In a previous published study from our research group, we obtained a specimen set from dogs histopathologically diagnosed with hemangiosarcoma (HSA), and determined that the prevalence of *Bartonella* spp. DNA (indicative of *Bartonella* infection in these dogs) was remarkably high (73%) [47]. The availability of multiple specimen types (serum, blood, and fresh frozen tissue biopsies) from each dog, as well as the overall high proportion of dogs with evidence of *Bartonella* spp. infection, made this a uniquely useful sample set in which to compare results from two newly developed assays (WB, ddPCR) to multiple commonly performed and commercially available assays (IFA, qPCR), in both blood and tissue samples. The objective of this study was to estimate clinical sensitivity and specificity of these commonly performed and newly-reported diagnostic assays for detection of *Bartonella* spp. across multiple sample types. Our hypothesis was that WB and ddPCR would be more sensitive for detection of *Bartonella* antibodies or DNA in blood and tissue samples from naturally infected dogs, when compared to IFA and qPCR assays.

## 2. Results

**Figure 1:**
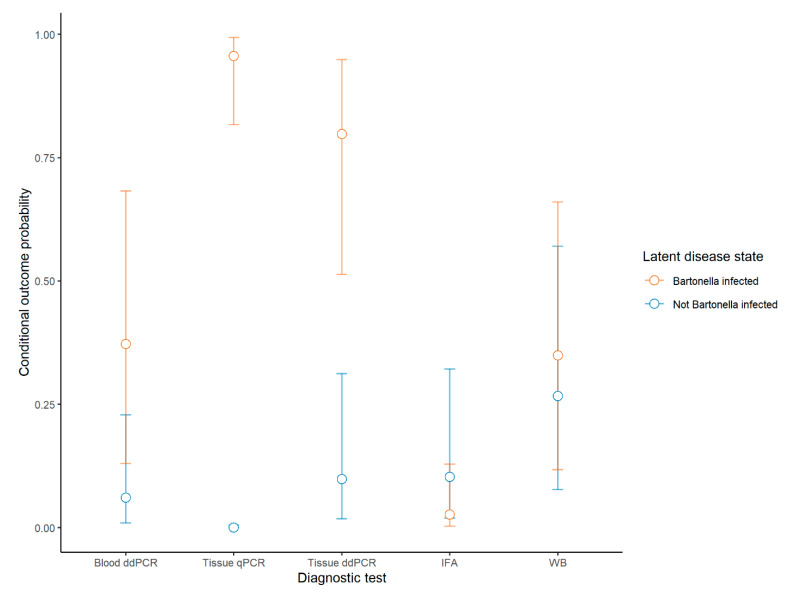
Conditional outcome probabilities of a positive result for class (*Bartonella* infected, in orange or Not *Bartonella* infected, in blue) for each test. The conditional outcome probability is the probability of a positive result for a subject with a random effect of zero (an “average” subject). Points show the probability estimate calculated based on RE-LCA, and bars show the 95% confidence interval for the estimate.

There were 90 dogs included in this study, with a median age of 10 years (range 4–20 years); 51% of the dogs were female, and the most common breeds were mixed breed (24), Labrador retriever (17), and Golden retriever (11), with other breeds each representing ≤5% of the study group. When results from all six assays (IFA, WB, qPCR on blood, qPCR on tissue, ddPCR on blood, and ddPCR on tissue) were evaluated in parallel, *Bartonella* DNA and/or antibodies were found in 70 of the 90 dogs (78%). There were 59 dogs (66%) that had a positive result on any two or more assays. The serology and PCR results for each of the six assays are shown in Table 1. The RE-LCA and CRS estimated sensitivity and specificity for each assay are shown in Table 1. Though not clinically practical, to maximize the sensitivity of detection of *Bartonella* spp. an inclusive CRS was defined for sensitivity calculations using the combined results of all six assays in parallel: a dog was considered positive on the inclusive reference standard if it had a positive result on any one or more of these six assays, and considered negative it was negative on all six assays. Since specificity could not be calculated with this inclusive CRS, a separate CRS was defined for calculation of specificity of each test: for the specific CRS, a dog was considered positive if it had a positive result on any two or more of the six assay, and considered negative it was negative on all six assays or positive on only one assay. Based on RE-LCA, 64% of dogs were classified as infected. The conditional probability of a dog being classified as *Bartonella* infected or not *Bartonella* infected, based on RE-LCA, for each test is shown in Figure 1.

In this group of dogs with HSA, the single assay with the highest sensitivity was qPCR performed on fresh frozen tissue biopsy samples (94% [95% CI 79–100%]). This assay also had the highest estimate specificity (100% [95% CI 71–100%]). For each individual assay, Table 1 also reports the number of dogs that were only positive on the respective assay, but negative by the remaining five assays. The Tissue qPCR had the highest sensitivity with the lowest false positive rate. While tissue ddPCR, blood ddPCR, and IFA had similar false positive rates, they differed widely in sensitivity. The observed and expected frequencies of each combination of assay results, along with the probability that a dog with each combination of results would be classified as infected by the RE-LCA model, are shown in Table 2.

When comparing newly-reported to traditional *Bartonella* diagnostic assays, ddPCR was more sensitive than qPCR for detection of *Bartonella* DNA when testing blood samples (36% vs. 0%, *p* < 0.0001), though it was not when testing tissue samples (71% vs. 80%, *p* = 0.324). Based on the kappa value, there was moderate agreement between qPCR and ddPCR when both assays were performed on tissue biopsy samples (kappa = 0.59, Table 3). Tissues from 12 dogs were qPCR positive but ddPCR negative (i.e., 21% of the qPCR positive tissues were ddPCR negative), compared to tissues from only six dogs that were ddPCR positive but qPCR negative (i.e., 12% of the ddPCR positive tissues were qPCR negative). Based on the RE-LCA model, a dog with a positive result on tissue qPCR had a >99% probability of being classified as infected, regardless of the other assay results (positive or negative). Based on the kappa value, there was no to slight agreement between WB and IFA when both assays were performed on serum samples (kappa = 0.13, Table 4). The combined results from the two most commonly used assays taken in parallel (serum IFA and blood qPCR) had 9% sensitivity when compared to the reference standard.

When comparing combined molecular results with combined serologic results, molecular testing was more sensitive for detection of *Bartonella* spp. (molecular testing 91% sensitivity, serology 46% sensitivity, *p* < 0.0001). A dog was considered molecular assay positive if *Bartonella* spp. DNA was PCR amplified from one or more blood or tissue sample by qPCR, ddPCR, or both. A dog was considered serologically positive if it was IFA seroreactive and/or WB positive. When comparing overall *Bartonella* spp. molecular results with serologic results, there was slight to no agreement (kappa = 0.13, Table 5). Of 64 dogs with *Bartonella* DNA amplified from blood or tissue, only 41% (26 dogs) were seroreactive. Conversely, 19% of the 32 seroreactive dogs did not have DNA amplified from blood or tissue. Based on the RE-LCA model, dogs that were positive on serology alone (WB only, or WB and IFA) with negative molecular assays were highly unlikely (<3%) to be classified as infected.

When comparing combined molecular assay results between blood and tissue samples, tissue molecular testing was more sensitive for the detection of *Bartonella* spp. DNA (blood molecular sensitivity 36%, tissue molecular sensitivity 89%, *p* < 0.0001). A dog was considered tissue molecular test positive if *Bartonella* spp. DNA was PCR amplified from one or more tissue biopsy samples using qPCR, ddPCR, or both (each dog had two tissue biopsy samples tested). A dog was considered blood molecular test positive if *Bartonella* spp. DNA was PCR amplified from its blood sample using qPCR, ddPCR, or both. Based on the kappa value, there was fair agreement between the molecular assay results when performed on blood samples compared to tissue samples (kappa = 0.22, Table 6). Of 62 dogs with *Bartonella* spp. DNA PCR amplified from tissue samples, only 37% (23 dogs) also had *Bartonella* DNA PCR amplified from blood samples. The combined result from three assays taken in parallel (WB, tissue qPCR, and tissue ddPCR) had 100% sensitivity when compared to the inclusive reference standard.

*Bartonella* species could be identified with Sanger sequencing of PCR products from qPCR. Since no dog had *Bartonella* spp. DNA amplified from a blood sample by qPCR, *Bartonella* spp. identity could only be determined from tissue biopsy samples. In these tissue biopsies from dogs with HSA, *B. henselae* DNA was most often amplified. Homologies ranged from 99.3% to 100% (of the 138 bp analyzed) with *B. henselae* CAL1 and SA2 (Genbank accessions AF369527 and AF369529, respectively). Of the 56 *Bartonella* qPCR positive tissues, 52 (93%) contained *B. henselae* DNA, including 1 dog that had both *B. henselae* and *B. koehlerae* DNA PCR amplified from tissue. One dog had only *B. koehlerae* DNA PCR amplified from tissue (140/140 bp, 100% homology with Genbank accession AF312490). In two dogs, the *Bartonella* species could not be determined for the DNA sequences amplified; for these two samples, homology ranged from 95% to 98% with either *B. henselae* CAL1 or *B. henselae* SA2.

Two biopsy samples were tested with each molecular assay. There were 12 dogs that had *Bartonella* spp. DNA ddPCR amplified from both biopsy samples and 38 that had *Bartonella* spp. DNA ddPCR amplified from one of the two biopsy samples. There were 15 dogs that had *Bartonella* spp. DNA qPCR amplified from both biopsy samples (of these, 9 had the same species and strain, based on Sanger sequencing of PCR products), and 41 that had *Bartonella* spp. DNA qPCR amplified from one of the two biopsy samples.

## 3. Discussion

In this study estimating sensitivity and specificity of *Bartonella* spp. diagnostic assays, 62% of dogs with histopathologically confirmed HSA and multiple *Bartonella* spp. assays performed were classified as infected with >99% probability based on latent class analysis. *Bartonella* qPCR performed on fresh frozen tissue biopsy samples had the highest estimated sensitivity and specificity (94% and 100% respectively) of any single assay. A dog with a positive result on tissue qPCR had a >99% probability of being classified as infected, regardless of the other assay results (positive or negative).

When comparing newly-reported to traditional *Bartonella* diagnostic assays, ddPCR was not more sensitive for detection of *Bartonella* DNA than qPCR when testing tissue samples (78% vs. 94% sensitivity). Though 70 dogs in this study had molecular and/or serological evidence of exposure to or infection with a *Bartonella* spp. when tested with all six different assays, not a single dog had *Bartonella* spp. DNA amplified from blood by qPCR, and only 8% were seroreactive by IFA. Based on RE-LCA, four of the six IFA positive dogs had >94% probability of being classified as not infected, demonstrating that while IFA is more specific than sensitive, the specificity is unlikely to be perfect. In contrast, half of the dogs positive by WB were classified as not infected–these included mainly dogs that were positive by WB alone or by WB and IFA (but negative on all molecular assays).

While qPCR did not amplify *Bartonella* DNA from any dog’s blood sample, ddPCR proved a more sensitive DNA detection method from the blood. However, even ddPCR performed on blood samples had an estimated sensitivity of only 38%. There were 39 dogs in this study that had *Bartonella* DNA amplified from their tissue samples, but both ddPCR and qPCR testing of blood were negative. Previous studies in dogs and humans have shown very good analytical accuracy and high specificity of well-designed PCR assays [24,46,64]. Despite this, the discrepancy between PCR amplification of DNA from blood and tissue samples is also commonly reported in recent studies [47,48,49,51]. Recent examples include an experimental study in which bacteremia was never confirmed in dogs experimentally infected with *Bartonella* spp., despite viable *Bartonella* spp. being isolated from tissues (bone marrow and lung) collected at the time of post-mortem examination [51]. In another clinically relevant report, a dog had *B. henselae* DNA PCR amplified from biopsies of vasculitis lesions and normal skin, but *Bartonella* DNA was not PCR amplified, even after BAPGM enrichment culture, from multiple sequential blood samples [65].

Proposed explanations for lower sensitivity of both qPCR and ddPCR in blood compared to tissue include both the variable nature of *Bartonella* spp. bacteremia, as well as the stochastic and dilutional phenomena associated with small quantities of *Bartonella* spp. DNA in patient blood samples. DdPCR was specifically designed to detect low-copy-number DNA on a background of abundant host DNA, and there were 25 dogs in this study that had qPCR negative but ddPCR positive blood samples. This supports the theory that small quantities of *Bartonella* spp. DNA in blood samples limits the clinical sensitivity of qPCR; ddPCR appears to improve upon this limitation. Testing multiple blood samples per dog (similar to the “triple draw” technique recommended for blood sampling for *Bartonella* PCR/BAPGM in humans) [66] might further increase the diagnostic sensitivity of both qPCR and ddPCR performed on blood samples, though this has not been specifically evaluated previously in dogs. In addition, BAPGM enrichment culture on blood samples might improve sensitivity [45,46], but the blood samples provided for this study were not collected or processed aseptically so enrichment culture was not possible. The sampling protocol used in this study may be another explanation for the higher sensitivity of molecular testing performed on tissues compared to blood samples. Because of the originally determined sampling protocol for the CCOGC, dogs had two biopsy samples obtained and submitted. Considering tissue results using two samples likely increased the sensitivity of this assay compared to using only one sample for molecular testing of blood. Indeed, there were only nine dogs for which the same *Bartonella* spp. DNA was amplified by qPCR from both tissue biopsy samples.

Adding to the difficulty interpreting the clinical significance of *Bartonella* bacteremia, as indicated by PCR positive blood samples, *Bartonella* spp. bacteremia can also be fairly common in apparently healthy dogs. One study of healthy volunteer dogs in North Carolina found that approximately 10–20% had evidence of *Bartonella* spp. bacteremia based on BAPGM enrichment blood culture/PCR [67]. The reasons that *Bartonella* DNA is not cultured or PCR amplified from blood samples from dogs with *Bartonella* DNA present in tissue could include intermittent bacteremia, rapid clearance of bacteremia by the dogs’ immune system, a transient bacteremic phase of infection prior to a chronic tissue phase, or lower concentrations of bacterial DNA in blood compared to tissue. Regardless of the mechanism(s), *Bartonella* PCR performed on a single blood sample does not effectively predict whether a dog has *Bartonella* spp. DNA in its tissues.

It is important to emphasize that fresh frozen rather than formalin fixed paraffin embedded (FFPE) tissues were used for the molecular assays performed in this study. Formalin fixation decreases PCR sensitivity by making DNA extraction more difficult, mainly because of chemical modification, as well as DNA trapping and potentially DNA fragmentation [68]. Though head-to-head comparisons of *Bartonella* spp. ddPCR on fresh frozen compared to FFPE tissues have not been reported, based both on the mechanism by which formalin fixation decreases qPCR sensitivity, as well as studies of other infectious diseases, it is reasonable to assume that ddPCR sensitivity is also decreased when performed on FFPE tissue samples compared to fresh frozen samples [69]. Thus, it is of critical importance that clinicians collect and store frozen tissue at the time of biopsy, surgery, or autopsy if PCR-based molecular diagnostics are to be performed.

While we were fortunate to have a set of samples for this study that included two biopsy samples of tissue as well as blood and serum, obtaining tissues is an invasive procedure that carries risks for complications and is generally accompanied by substantial sampling expense. These practical constraints may limit the widespread clinical utility of tissue biopsies for routine *Bartonella* testing. However, it is notable that many of the tissues included in this study were skin biopsies from normal-appearing skin in dogs with pathology in distant organs. Skin biopsies can in fact often be obtained at substantially less risk and expense than biopsies of other potentially infected organs (spleen, liver, heart). The distribution of *Bartonella* in tissues is not likely uniform, but given the ability for *Bartonella* to induce pathology systemically via secreted factors such as BafA, and to infect vascular endothelial cells which are distributed throughout the body, it is possible that *Bartonella* infection in the skin may ultimately be shown to correlate with systemic illness [70,71]. However the importance and variability of the localization of *Bartonella* infection in any given organ is poorly characterized at this time [70].

In regard to *Bartonella* serology, when both WB and IFA results were considered in parallel *Bartonella* spp. serology was positive in less than half (36%) of HSA dogs that had *Bartonella* spp. DNA amplified from blood or tissue. Based on the RE-LCA model, dogs that were positive on serology alone (WB only, or WB + IFA) with negative molecular assays were highly unlikely (<3%) to be classified as infected. Previous studies documenting poor agreement between *Bartonella* spp. IFA seroreactivity and *Bartonella* spp. bacteremia based on PCR/BAPGM in dogs have been reported [44,46,72]. IFA is known to have low sensitivity and may underestimate the true seroprevalence of *Bartonella* spp. exposure in dogs [44,46,73,74,75]. IFA in this group of dogs with HSA had a lower sensitivity and specificity (4% and 88% respectively) than has been historically reported, with one early study estimating *Bartonella* spp. IFA sensitivity at approximately 40% when compared to documentation of active infection by BAPGM enrichment blood culture/PCR [46], and a more recent study estimating a 62% sensitivity when using an expanded IFA panel including eight *Bartonella* spp. antigens, again compared to positive BAPGM enrichment blood culture/PCR results [44]. Possible explanations for the lower IFA sensitivity reported here may be due to immune evasion or modulation at different stages of infection, since previous reports have investigated BAPGM enrichment blood culture/PCR rather than tissue biopsy samples, and blood samples do not always accurately reflect the status of tissue infection. Since none of the dogs in this report had *Bartonella* spp. DNA amplified from blood samples using qPCR, we cannot evaluate the sensitivity/specificity of IFA in dogs positive only by qPCR performed on blood samples.

WB had higher sensitivity compared to IFA for detecting *B. henselae* antibodies (36% vs. 4%), but also lower specificity and a higher false positive rate. The five dogs positive solely by WB had a 97% probability of being classified as not infected based on the RE-LCA model (false positives). Because it is not possible to distinguish whether the antibodies detected by WB or IFA are indicative of active infection or previous exposure with effective clearance of bacteria, it is possible that seroreactive dogs were previously exposed, but not actively infected with a *Bartonella* spp. Conversely, for the 38 dogs that had *Bartonella* spp. DNA PCR amplified from blood and/or tissue, but were nonseroreactive, it is possible that the bacterial strategies used to evade the immune system in intracellular infection contribute to a lack of detectable antibodies by either IFA or WB.

This study was designed to be primarily descriptive, and as such has multiple limitations. Importantly, there was no control group consisting of confirmed *Bartonella*-negative dogs with which to evaluate test specificity in similar manner to the traditional method using the inclusive reference standard to estimate sensitivity, and therefore ground our RE-LCA model in observed data. Using RE-LCA precludes the need for “known” disease status, however, and allows estimation of both sensitivity and specificity without a control population. Previous studies attempting to identify populations of dogs that had never been exposed to, or infected with, *Bartonella* spp. to serve as controls have often encountered considerable difficulty. For example, in an early study attempting to develop an experimental infection model of *B. henselae* and *B. vinsonii* subsp. *berkhoffii*, the two dogs obtained from a university breeding facility were infected with *B. koehlerae* prior to initiation of the study [51]. This, and other studies showing *Bartonella* infection or exposure in dogs expected to be unexposed/uninfected, illustrate the difficulty of finding a truly negative dog population with which to evaluate test specificity [49,67]. In an attempt to minimize false negatives, we included molecular assay results on both of two distinct tissue biopsies submitted or each dog; this likely increased the sensitivity of molecular assays performed on tissue compared to blood samples (of which only one sample per dog was tested). Future studies evaluating diagnostic accuracy metrics for *Bartonella* spp. assays should use the same number of specimens for each sample type, and consider evaluating multiple blood samples to determine the degree to which repeated sampling improves molecular assay sensitivity in this sample type. In addition, future studies could consider using Bayesian techniques to evaluate diagnostic accuracy of *Bartonella* spp. assays. LCA using a Bayesian framework has been successfully performed for decades, and allows for greater flexibility in model creation as well as the use of informed priors that could be based on this and other studies [76,77,78].

Another limitation of this study concerns using PCR (qPCR or ddPCR) as a diagnostic method, because demonstration of pathogen DNA in a patient sample does not necessarily mean that live/viable pathogen is present in the patient, nor that that pathogen is the cause of the patient’s clinical disease [79]. For *Bartonella* spp., demonstration of live/viable bacteria is possible with culture or BAPGM-ePCR; unfortunately the samples in this study were not collected sterilely, precluding BAPGM-ePCR. Finally, because this was a retrospective study using a previously obtained set of samples from dogs with hemangiosarcoma, the results of this study cannot be applied to the diagnosis of other clinical or pathological disease presentations in dogs.

Currently, the “gold standard” of bartonellosis diagnosis (in dogs or humans) continues to rely on a combination of culture, serology, and molecular tests with poor, variable, or undetermined sensitivity, specificity, or both. In the absence of a true gold standard, it is difficult to accurately estimate clinical sensitivity and specificity of diagnostic tests. Without this information, it is impossible for clinicians to critically interpret test results, for epidemiologists to draw appropriate conclusions about population-wide trends, or for vector biologists and ecologists to clarify modes and routes of transmission. When clinical diagnostic tests are assumed to be more sensitive than they are, false negatives are not recognized and serious or fatal cases of bartonellosis may go undiagnosed [24,80]. False positives may also lead to inaccurate diagnosis and potentially inappropriate treatments, particularly with unnecessary antibiotics. Our findings emphasize the need to further investigate the sensitivity and specificity of serological and molecular diagnostic tests for *Bartonella* spp.—both those assays currently in commercial use and novel assays that are continuing to be developed–in a variety of clinical settings. These findings also illustrate the limitations and diagnostic complexity associated with determination of *Bartonella* spp. exposure and infection in clinical patients, particularly as IFA and blood qPCR–the two most common diagnostic assays used by clinicians–had poor diagnostic accuracy. As many epidemiology studies are also based solely on these assays, the lack of sensitivity of these assays illustrated in this and other studies should be considered by researchers attempting to define *Bartonella* disease manifestations, ecology, transmission, and zoonotic risks.

## 4. Methods

### 4.1. Study Design and Sample Sources

This was a retrospective, observational, descriptive study involving 90 dogs with HSA. Specimens used for this study were previously collected from eight university veterinary hospitals across the United States between May 2008 and November 2011 and were banked by the Canine Comparative Oncology and Genomics Consortium (CCOGC) based on previously published standard operating procedures. [81] These samples were used in a previous study from our laboratory to determine the proportion of dogs with HSA that had hemotropic pathogen DNA (*Babesia, Bartonella,* and hemotropic *Mycoplasma* spp.) in their blood or tissues [47]. The CCOGC provided demographic information for each dog, including age (years), breed, weight (kg), and sex and neuter status. The date and geographic location (university veterinary hospital) of sample collection was also provided. The anatomic location and histopathological diagnosis of HSA for each dog was also provided, and confirmed as previously described [47].

There were 110 eligible dogs from the originally published study [47]; of these, 20 dogs were missing one or more sample type and were therefore excluded, resulting in complete specimen set from 90 dogs that were ultimately used to generate the results reported in this study. Three specimen types were defined for pathogen testing: whole blood, serum, and fresh frozen tissue biopsies. For tissues, two biopsy samples were tested for each dog. The majority of tissues submitted were biopsies from splenic HSA tumors (64), histopathologically normal spleen (27), and histopathologically normal skin and/or adipose tissue (29); other tissues (60) included cardiac (HSA tumor and nontumor tissue), skeletal muscle, liver (HSA tumor and nontumor tissue), lung, kidney, mammary gland, and tissues of undetermined origin.

### 4.2. Bartonella Detection Methods

Traditional diagnostic tests for *Bartonella* spp., including IFA and qPCR on blood and tissue, were performed as part of the previous study [47]. For IFA testing, *Bartonella* antibodies were determined using three *Bartonella* spp. grown in cell culture (*Bartonella henselae*, *Bartonella vinsonii* subsp. *berkhoffii*, and *Bartonella koehlerae*) as antigens and following standard immunofluorescent antibody assay (IFA) techniques [47]. Sera were first screened at dilutions of 1:16 to 1:64. All sera that are reactive at 1:64 were further tested with two-fold dilutions to 1:8192. A dog was considered IFA seroreactive if it was reactive at ≥1:64 against any one or more of the three *Bartonella* spp. antigens. For qPCR, each sample was screened for the presence of *Bartonella* spp. DNA using primers targeting the 16S-23S intragenic transcribed spacer (ITS) region of *Bartonella* spp., using 5 μL of template DNA as previously described [47,82]. Validation of positive qPCR results was performed by Sanger sequencing of amplicons followed by chromatogram evaluation and sequence alignment using Contig-Express and Align X software (Vector NTI Suite 10.1, Invitrogen Corp, Carlsbad, CA, USA). For bacterial species identification, DNA sequences were analyzed for nucleotide sequence homology at NCBI nucleotide database using BLAST version 2.0. A dog was considered *Bartonella* spp. qPCR positive on tissue if one or more of the biopsy samples was qPCR positive (biopsy samples run in parallel). Stringent processing methods were used to avoid DNA carryover during tissue processing [47,58,83].

For the current study, each DNA sample was screened for the presence of *Bartonella* spp. DNA using ddPCR with the same primers and probes employed for qPCR and minor modifications [58]. The procedure was amended and validated for dogs as described for human testing [58]. DNA extractions from *B. henselae*, *B. quintana*, *B. koehlerae* or *B. vinsonii subsp. berkhoffii* genotype II spiked into negative control dog blood samples (at a concentration of 0.5 bacteria/μL) were used to set threshold values and determine the limit of detection. ddPCR amplification of the dog housekeeping gene was also performed using primers and probes employed for qPCR, as a reference target to facilitate quantification. Due to instrument design limitations, digital PCR droplets are not able to be sequenced. WB was performed as previously described using *B. henselae* SA2 whole-cell protein lysates and goat antidog whole IgG [56]. As previously validated, a dog was considered WB positive if seroreactive to any two or more *B. henselae* immunodominant proteins with a molecular size of 13, 17, 29, 50, 56, and 150 kDa (allowing for a ±1 kDa margin of error in interpretation) [56].

### 4.3. Study Size and Statistical Methods

Data analysis was performed using R 4.0.2 (https://www.R-project.org/, accessed on 25 June 2020). The percentage of dogs positive for each individual assay, as well as different combinations of assays, were calculated. Diagnostic test results were presented in contingency tables. In the absence of an accepted gold standard for diagnosis of bartonellosis in dogs, two methods were used to estimate diagnostic test parameters: latent class analysis with random effects (RE-LCA) and composite reference standard (CRS) techniques.

In LCA models, the true disease status is unobserved (the so-called latent variable) and diagnostic parameters are estimated based on this latent state. Using this method, a single gold standard diagnostic test is not needed, but rather each test is assumed to be imperfect and the probability of a patient being classified into each disease state is determined based on the observed combination of diagnostic test results. Classical LCA is based on the assumption of conditional independence; that is, test results for each subject are assumed to be independent, conditional on the latent state. This assumption is not necessarily met for the patients in this study: conditional independence of IFA and WB cannot be assumed, since both are based on detection of antibodies, and conditional independence of ddPCR and qPCR also cannot be assumed, since both use the same gene targets. There may also be heterogeneity between patients that is not dependent on their classification (infected or noninfected), for example based on infection severity, location, or duration. Therefore LCA with random effects was performed to account for heterogeneity among patients within each classification, with a normally distributed random effect added for each subject. RE-LCAwas done using the “randomLCA” package in R. [84] The five assays described above were used as observed indicators. Random effects LCA models using equal loading and probit scaling of the random effect were created. To investigate the possibility that *Bartonella* infection is not a binary outcome (infected vs. not infected), models with one, two, and three latent classes were compared. The number of quadrature points was left at default (quadpoints = 21). Model selection was based on BIC [84]. The minimum BIC was obtained using the model with two latent classes, and this model was therefore selected. Based on this model, classification as “*Bartonella* infected” or “Not *Bartonella* infected” was determined with membership probabilities for each class computed from the estimated model parameters. Sensitivity and specificity (and their 95% confidence intervals) were calculated based on marginal outcome probabilities.

The use of a composite reference standard (CRS) is an alternative method by which to assess test accuracy using a combination of imperfect tests [62,63,85,86]. For this study, an inclusive CRS was defined for sensitivity calculations in order to maximize the sensitivity of detection of *Bartonella* spp. infection/exposure and decrease the likelihood of false negatives. The inclusive CRS was defined using the combined results of all six assays in parallel: a dog was considered positive on the reference standard if it had a positive result on any one or more of these six assays, and considered negative it was negative on all six assays. Since specificity could not be calculated with this inclusive CRS, a separate CRS was defined for calculation of specificity of each test: for the specific CRS, a dog was considered positive if it had a positive result on any two or more of the six assay, and considered negative it was negative on all six assays or positive on only one assay. For the purpose of this study, a dog was considered to have evidence of *Bartonella* spp. infection if it had *Bartonella* spp. DNA PCR amplified on one or more assay, and considered to have *Bartonella* spp. exposure if it was IFA seroreactive or WB positive. With 90 dogs, we were able to estimate 95% confidence intervals (CIs) for the sensitivity of each test within a margin of ±approximately 11% These CIs for the sensitivity of each test were calculated using the Wilson score interval. *Bartonella* spp. positive proportions were compared using chi-squared tests with continuity correction. Statistical significance was considered when *p* < 0.05. To determine agreement between tests, the kappa statistic was calculated [87].

## Figures and Tables

**Table 1:** PCR and serology results for each of six different *Bartonella* diagnostic assays. The table shows the number of dogs positive by each assay, sensitivity and specificity of each assay estimated from RE-LCA, sensitivity of each assay or combination of assays compared to the CRS defined for this study, and the number of dogs positive on each individual assay but negative on the remaining five assays. The inclusive reference standard was defined for this study as a combination of results from all six assays evaluated in parallel.

	Positive Dogs (*n* = 90)	% Sensitivity (95% CI)		% Specificity (95% CI)		Dogs Positive Solely by This Method
		RE-LCA	CRS	RE-LCA	CRS	
*Individual molecular tests*						
Blood qPCR	0	--	0 (0–5.2)	--	NA	0
Blood ddPCR	25	38 (25–54)	36 (26–47)	92 (80–100)	100 (89–100)	0
Tissue ddPCR	50	78 (65–98)	71 (60–81)	88 (70–100)	94 (79–98)	2
Tissue qPCR	56	94 (79–100)	80 (69–88)	100 (71–100)	87 (71–95)	4
*Individual serology tests*						
IFA	6	4 (0–9)	8.6 (4.0–18)	88 (72–100)	100 (89–100)	0
WB	30	36 (23–49)	43 (32–55)	72 (50–89)	84 (67–93)	5
*Combinations of tests*						
Any serology test	32	--	46 (35–57)	--	84 (67–93)	--
Any molecular test	64	--	91 (83–96)	--	81 (64–91)	--
Tissue qPCR + IFA (in parallel)	60	--	86 (76–92)	--	87 (71–95)	--
Any of the six assays (CRS sensitivity)	70	--	*reference*	--	65 (47–79)	--
2 or more of the six assays (CRS specificity)	59	--	84 (74–91)	--	*reference*	--

**Table 2:** Results of RE-LCA model. For each combination of assay results observed in the dataset, the number of dogs is shown. Observed indicates the number of dogs with the specified pattern counted in the dataset. Expected indicates the number dogs with the specified pattern predicted by the RE-LCA model. The probability of being classified as infected or not infected, as predicted by the RE-LCA model, for a dog with each specified pattern, is also shown. For each assay result, a 1 indicates a positive result and a 0 indicates a negative result.

	Assay Result					Number of Dogs		Probability of Classification	
Result summary	Blood ddPCR	Tissue qPCR	Tissue ddPCR	IFA	WB	Observed	Expected	Infected	Not infected
Blood ddPCR + Tissue qPCR + Tissue ddPCR only positive	1	1	1	0	0	11	9.7	1.0	0.0
All positive except IFA	1	1	1	0	1	6	7.3	1.0	0.0
Tissue qPCR + Tissue ddPCR only positive	0	1	1	0	0	16	16.4	1.0	0.0
Tissue qPCR + Tissue ddPCR + WB only positive	0	1	1	0	1	9	8.8	1.0	0.0
Blood ddPCR + Tissue qPCR only positive	1	1	0	0	0	3	2.4	1.0	0.0
Blood ddPCR + Tissue qPCR + WB only positive	1	1	0	0	1	1	1.3	0.999	0.001
Tissue qPCR + Tissue ddPCR + IFA only positive	0	1	1	1	0	1	0.5	0.999	0.001
Tissue qPCR only positive	0	1	0	0	0	4	5.9	0.999	0.001
All positive except blood ddPCR	0	1	1	1	1	1	0.4	0.998	0.002
Tissue qPCR + WB only positive	0	1	0	0	1	4	2.2	0.998	0.002
Blood ddPCR + Tissue ddPCR only positive	1	0	1	0	0	2	0.6	0.720	0.280
Tissue ddPCR only positive	0	0	1	0	0	2	2.9	0.410	0.590
Blood ddPCR + WB only positive	1	0	0	0	1	1	0.6	0.099	0.901
Tissue ddPCR + IFA only positive	0	0	1	1	0	1	0.3	0.059	0.941
Tissue ddPCR + IFA + WB only positive	0	0	1	1	1	1	0.2	0.044	0.956
All assays negative	0	0	0	0	0	20	17.4	0.037	0.963
WB only positive	0	0	0	0	1	5	5.9	0.027	0.973
Blood ddPCR + IFA + WB only positive	1	0	0	1	1	1	0.1	0.010	0.990
IFA + WB only positive	0	0	0	1	1	1	0.9	0.003	0.997

**Table 3:** Comparison between qPCR and ddPCR when testing tissue biopsy samples from dogs with HSA. Number and percentage of all dogs (*n* = 90) with positive or negative result for each assay shown.

		Tissue qPCR Result	
		Positive	Negative
**Tissue ddPCR result**	Positive	44 (49%)	6 (6.7%)
	Negative	12 (13%)	28 (31%)

**Table 4:** Comparison between WB and IFA when testing serum samples from dogs with HSA. Number and percentage of all dogs (*n* = 90) with positive or negative result for each assay shown.

		IFA Result	
		Positive	Negative
**WB result**	Positive	4 (4.4%)	26 (29%)
	Negative	2 (2.2%)	58 (64%)

**Table 5:** Comparison between Bartonella serological and molecular assay results for 90 dogs di-agnosed with HSA. Molecular assays included qPCR and ddPCR on blood and tissue; serologic assays included IFA and WB. Number and percentage of all dogs (*n* = 90) with positive or negative result for each combination of assays shown.

		Molecular Result	
		Positive	Negative
**Serology result**	Positive	26 (29%)	6 (6.7%)
	Negative	38 (42%)	20 (22%)

**Table 6:** Comparison between molecular testing on blood and tissue. A positive tissue molecular test was defined as a dog that had Bartonella DNA amplified from one or more tissue samples using qPCR, ddPCR, or both. A positive blood molecular test was defined as a dog that had Bartonella DNA amplified from one or more blood samples using qPCR, ddPCR, or both. In this sample of 90 dogs with HSA, all blood qPCR results were negative.

		Tissue Molecular Result	
		Positive	Negative
**Blood molecular result**	Positive	23	2
	Negative	39	26

## Data Availability

Data is available via Dryad (https://doi.org/10.5061/dryad.h18931zm1 accessed on 25 June 2020).

## Abbreviations

hemangiosarcoma (HSA), composite reference standard (CRS), latent class analysis (LCA), random effects latent class analysis (RE-LCA), immunofluorescence assay (IFA), western immunoblot (WB), droplet digital PCR (ddPCR), quantitative PCR (qPCR), cat scratch disease (CSD), Bartonella alpha-proteobacteria growth medium (BAPGM), formalin-fixed paraffin embedded (FFPE), Canine Comparative Oncology and Genomics Consortium (CCOGC), intragenic transcribed spacer (ITS), 95% confidence interval (95% CI)
